# Roaming pathways and survival probability in real-time collisional dynamics of cold and controlled bialkali molecules

**DOI:** 10.1038/s41598-021-90004-0

**Published:** 2021-05-19

**Authors:** Jacek Kłos, Qingze Guan, Hui Li, Ming Li, Eite Tiesinga, Svetlana Kotochigova

**Affiliations:** 1 Department of Physics, Temple University, Philadelphia, PA 19122 USA; 2 Joint Quantum Institute, University of Maryland, College Park, MD 20742 USA; 3 National Institute of Standards and Technology, Gaithersburg, MD 20899 USA

**Keywords:** Chemistry, Physical chemistry

## Abstract

Perfectly controlled molecules are at the forefront of the quest to explore chemical reactivity at ultra low temperatures. Here, we investigate for the first time the formation of the long-lived intermediates in the time-dependent scattering of cold bialkali $^{23}\hbox {Na}^{87}$Rb molecules with and without the presence of infrared trapping light. During the nearly 50 nanoseconds mean collision time of the intermediate complex, we observe unconventional roaming when for a few tens of picoseconds either NaRb or $\hbox {Na}_2$ and $\hbox {Rb}_2$ molecules with large relative separation are formed before returning to the four-atom complex. We also determine the likelihood of molecular loss when the trapping laser is present during the collision. We find that at a wavelength of 1064 nm the $\hbox {Na}_2\hbox {Rb}_2$ complex is quickly destroyed and thus that the $^{23}\hbox {Na}^{87}$Rb molecules are rapidly lost.

## Introduction

The availability of dilute ultracold gases of neutral bialkali molecules in their absolute ground state^1–8^ and cold diatomic molecular ions^9–17^ is revolutionizing molecular science. For heteronuclear dimers this stems from their long-range and anisotropic dipole-dipole interactions. Specifically, molecules with tunable long-range interactions are an invaluable asset in the study of controlled chemical reactions^18–23^, while a gas of such molecules can be used to explore exotic many-body physics^24,25^. Moreover, their rotational states are excellent qubit states with natural entangling interactions^26–29^. These and other applications based on either ultracold heteronuclear or homonuclear molecules require control of their internal as well as external, motional degrees of freedom. This control is enabled by placing the molecules in laser-generated optical potentials, i.e. optical traps, lattices, or tweezers, where they can be detected and manipulated.

From a chemistry point of view two classes of ground-state alkali-metal molecules are relevant^30^. The first class are those molecules that are susceptible to exothermic chemical reactions, such as all heteronuclear dimers that contain a Li atom and $$^{40}\hbox {K}^{87}$$Rb^30–34^. Total reaction rate coefficients for the fermionic $$^{40}\hbox {K}^{87}$$Rb are on the order of $$10^{-12}\,\hbox {cm}^3$$/s for the current state-of-the-art nanokelvin temperatures when all molecules are prepared in the same spin state. Rate coefficients are on the order of $$10^{-10}\,\hbox {cm}^3$$/s otherwise. Other bialkali molecules are chemically stable with respect to molecule-molecule collisions. Examples are bosonic $$^{23}\hbox {Na}^{87}$$Rb^6,35^, $$^{23}\hbox {Na}^{39}$$K^8^, and $$^{87}\hbox {Rb}^{133}$$Cs^3,4,36^, and the fermionic $$^{23}\hbox {Na}^{40}$$K^5,7^. Their lifetime in optical traps was expected to be only limited by the rate of collisions with room-temperature molecules in the typical Ultra High Vacuum (UHV) environment used for these experiments and expected to be several seconds. This would, for example, make possible the simulation of novel many-body phases. Several recent experimental studies with these “stable” bosonic and fermionic molecules in optical traps, however, observed a short lifetime^3,5,35–38^ comparable to those of reactive molecules.

An explanation of this phenomenon, suggested by Ref.^39^, relies on the observation that electronically excited states of four-atom (tetramer) complexes become energetically accessible to resonant absorption of photons from the trapping lasers. This leads to uncontrolled spontaneous decay and loss of molecules from the trap. This loss mechanism was confirmed by recent experimental observations with $^{87}\hbox {Rb}^{133}$Cs^40^. In addition, the rate of product formation in the reactive collision of $^{40}\hbox {K}^{87}$Rb molecules was reduced by trapping light^2^.

The model of optical losses in collisions of fermionic NaK and KRb molecules in Refs.^2,39^ was based on a comparison of the potential energies of electronic ground and excited states of the four-atom complexes for a small set of geometries. Laser excitation rate coefficients were obtained as a phase-space averaged sum over excited states in time-independent perturbation theory.

The next logical step requires the time-dependent study of the quasi-classical collision dynamics of alkali-metal molecules, where the electronic character of the few-body complex can significantly change over only a few femtoseconds in synchrony with the motion of the nuclei. Moreover, the intermediate complex can be long-lived, i.e. last significantly longer then the characteristic rovibrational timescale of the reactants. Finally, the dynamics is typically barrierless^41^. As a result, the motion of the complex is expected to be chaotic, thoroughly mixing all motional degrees of freedom, leading to a statistical distribution of times between optical excitations and excitation probabilities, similar to the statistical distributions of the reaction products observed in Ref.^42^.

In this paper, we investigate for the first time the real-time non-reactive collision of cold millikelvin alkali-metal $^{23}\hbox {Na}^{87}$Rb molecules initialized in their absolute electronic and ro-vibrational ground state in the presence of continuous-wave (cw) infrared 1064-nm optical radiation that is offresonant for electronic excitations in the reactant dimers. Our theoretical model combines long-time quasi-classical trajectory (QCT) calculations using a global six-dimensional ground-state Born-Oppenheimer potential energy surface (PES) of $\hbox {Na}_2\hbox {Rb}_2$ derived from an analytic dimer-in-molecule (DIM) model^43^ with short-time QCT calculations using the ground electronic PES derived from *on-the-fly* density-functional-theory (DFT) calculations. The quantum nature of the $v=0$ vibrational and $J=0$ rotational ground state of $\hbox {X}^1\Sigma ^+$ NaRb molecules and their cold external motion are accounted for by micro-canonical sampling over initial phase-space points in position and momentum and computing the corresponding classical trajectories with Newton’s equations for the ground-state PES^44^. The relevant characteristics of the ground-state potential are described in Results and Methods.

The long-time QCT simulations with the easy-to-compute dimer-in-molecule PES have the primary goal of determining the probability distribution for the duration or lifetime of the intermediate tetramer complex but do not include laser radiation. These calculations are discussed in Results and have led us to the realization that there exist two types of trajectories. Those with lifetimes less than 1 ns are quasi-one dimensional, while those with lifetimes larger than 1 ns, corresponding to approximately 95% of all trajectories, undergo chaotic motion with mean collision time of $\approx 50$ ns. The lifetimes derived from these latter trajectories have Poisson statistics.

Section Results describes our discovery of pervasive *roaming* trajectories in $^{23}\hbox {Na}^{87}$Rb collisions that last more than 1 ns. Roaming trajectories or orbits correspond to temporary large-amplitude motion of the colliding atoms in a weakly-bound region of the tetramer potential. In our system roaming orbits correspond to the temporary formation of well-separated homonuclear $\hbox {Na}_2$ and $\hbox {Rb}_2$ molecules and of NaRb+NaRb with or without exchange of the two Na (or Rb) atoms. It is worth noting that, at our total energy, potential energies at conical intersections^45^ between the ground and first-excited $\hbox {Na}_2\hbox {Rb}_2$ states are endothermic by about $hc\times 500\,\hbox {cm}^{-1}$, where *h* is Planck’s constant and *c* is the speed of light in vacuum. Conical intersections are also discussed in Results.

By convention, roaming states only correspond to large-amplitude atom configurations that are substantially different from those obtained by transition-state reaction pathway theories^46^. Over the last twenty years, experimental and theoretical advances^47–51^ have made a convincing case for the presence of roaming dynamics. These studies, however, focussed on reactions with molecules containing hydrogen and other light atoms and were restricted to collision temperatures above one Kelvin^46,52–54^. Here, we have invigorated the research on roaming by exploring collisions of heavy molecules at sub-Kelvin temperatures.

**Figure 1:**
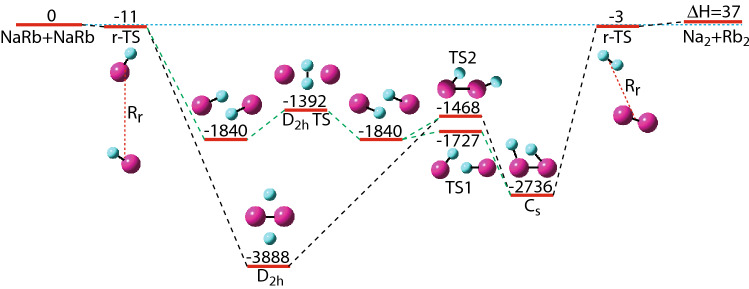
Energies of stationary points for the $^{23}\hbox {Na}_2^{87}\hbox {Rb}_2$ ground-state potential based on coupled-cluster calculations with all four atoms located in the same plane, i.e. have $C_s$ symmetry. All energies are given in units of $hc \times 1\,\hbox {cm}^{-1}$. The energy is zero for two $^{23}\hbox {Na}^{87}$Rb molecules at their equilibrium separation of the $\hbox {X}^1\Sigma ^+$ state. This state is shown on the left-hand side of the figure. The right-hand side shows the endothermic di-atomic $^{23}\hbox {Na}_2+^{87}\hbox {Rb}_2$ ground-state limit at the respective equilibrium separations. Other stationary states, minima and saddle points, in the PES (red horizontal lines) are shown in between. Saddle points are labeled by the abbreviations TS and r-TS corresponding to transition states and roaming transition states, respectively. For all stationary states a diagram shows the location of the four atoms. Na and Rb atoms are indicated by small cyan and large magenta spheres, respectively. Dashed black and blue lines connect stationary states and define reaction paths. Figure was prepared in Adobe Illustrator CS 6 with molecular structure pictures prepared using GaussView 5.09 for Mac.

The short-time QCT simulations are based on *on-the-fly* DFT calculations of the ground-state potential energy surface. Simultaneously, we use time-dependent density-functional-theory (TD-DFT) calculations to determine the splittings between excited- and ground-state potentials as well as electric transition dipole moments to include the effects of the trapping light. These simulations follow Ref.^55^ and are described in Results. Here, atoms move classically with forces determined by the DFT ground electronic potential and can be excited when the energy difference of the ground and excited states equals that of the energy of a photon in the trapping laser field.

Even when the DFT and TD-DFT potentials are only computed *on the fly* at the relevant classical positions of the atoms, the expense of electronic-structure calculations is prohibitive and the computations are limited to evolution times of about 10 ps, much less than needed to determine the duration of a typical collision. Even with this short evolution time, however, it became evident that the ground-to-excited-state energy difference is often equal to that of the energy of an infrared photon, typically with wavelength of 1064 nm. As a result, molecules moving on the ground-state collisional complex can be promoted to excited states and then lost from the trap. We applied the Landau-Zener model^56,57^ at each resonant geometry to determine an excitation probability. Specifically, for a 1064-nm photon we find that the collisional complex has on average optical excitations every 0.2 ps with a mean excitation probability of $2\times 10^{-5}$ at a typical laser intensity of 10 kW/$\hbox {cm}^2$. We have estimated the accuracy of the DFT and TD-DFT calculations with additional singles and doubles equation-of-motion coupled-cluster calculations (EOM-CCSD) at selected geometries along the classical paths.

The combination of the results from long- and short-time QCT simulations described in Results also enabled us to determine the likelihood that $^{23}\hbox {Na}^{87}$Rb molecules are broken up or destroyed by a laser during a collision. We find that this is extremely likely for trapping lasers at a wavelength of 1064 nm.

## Results

### Potential energy surface and reaction paths

We have performed non-relativistic coupled-cluster (CC) calculations with single, double, and non-iterative triple (CCSD(T)) excitations using the Karlsruhe def2-TZVPP basis set^58^ in order to find stationary points and reaction paths on the ground electronic state of $\hbox {Na}_2\hbox {Rb}_2$. We correlated all inner and valence electrons in the CC calculations. This state has a zero total electron spin. The CC calculations show that the stationary points predominantly occur when the four atoms are located at $C_s$ symmetries with all four atoms in a single plane.

Figure 1 shows the potential energies at stationary points with $C_s$ symmetry as well as the corresponding planar locations of the four atoms. The reactants NaRb+NaRb are shown on the left-hand side of the figure, while the products $\hbox {Na}_2$+$\hbox {Rb}_2$ are shown on the right-hand side. The product energy is only $hc\times 37\,\hbox {cm}^{-1}$ higher than that of the reactants and compares favorably with the spectroscopically determined value of $hc\times 47\,\hbox {cm}^{-1}$ derived in Ref.^35^. For the energetics of this system it is also worth realizing that the quantum-mechanical zero-point energy of the individual $\hbox {Na}_2$, NaRb, and $\hbox {Rb}_2$ dimers are $hc\times 80\,\hbox {cm}^{-1}$, $hc\times 53\,\hbox {cm}^{-1}$, and $hc\times 29\,\hbox {cm}^{-1}$ in their ground $\hbox {X}^1\Sigma _g^+$, $\hbox {X}^1\Sigma ^+$, and $\hbox {X}^1\Sigma _g^+$ state, respectively. This corresponds to a nearly identical combined zero-point energy of the reactants and products that is significantly larger than the $hc\times 37\,\hbox {cm}^{-1}$ endothermicity of our CC calculations. On the other hand, the difference between the combined zero-point energies is much smaller than the endothermicity.

Figure 1 further shows four minima. At the global minimum of the potential the atoms have $D_{2h}$ symmetry as well. $D_{2h}$ symmetry corresponds to planar geometries where the center of masses of the two Na atoms and of the two Rb atoms coincide and the Na-Na and Rb-Rb interatomic axes make a $90^\circ$ angle. The extrema have multiple equivalent atom geometries or configurations with the same potential energy reached by reflections through perpendicular planes or interchange of the Na atoms (or Rb atoms.) Only for the local minima at $hc\times -1840\,\hbox {cm}^{-1}$ relative to the reactant state, do we show equivalent states as those will be helpful in describing reaction paths below. Our potential energies at the minima agree well with the predictions in Ref.^59^.

The five saddle points or transition states in Fig. 1 separate out into three “conventional” planar transition states (TS) and two roaming transition states (r-TS). For both types of transition states the curvature of the potential along one or more normal-mode directions is negative, i.e. has a so-called imaginary frequency. Conventional or tight transition states have imaginary frequencies that are on the order of typical zero-point energies, here $\sim hc\times 100\,\hbox {cm}^{-1}$ from the dimer zero-point energies. Roaming transition states have at least one imaginary frequency that is much smaller than these typical values. Only one of the “conventional” transition states has $D_{2h}$ symmetry.

The two r-TS in our system have potential energies close to the reactant and product states and have geometries that closely resemble those of the reactant and product states. They are thus in the threshold regions of the PES. From Refs.^50,60^ we learned that such states can initiate dynamically distinct collisions. For $\hbox {Na}_2\hbox {Rb}_2$, these transition states will play an essential role in roaming collisional dynamics as discussed below. The only out-of-plane stationary point (except for equivalent geometries reached by identical atom interchange, reflections through the center of mass, etc.) is a second-order saddle point at an energy of ${hc}\,{\times}-1070\,\hbox {cm}^{-1}$ relative to the reactant state and has two directions along which the curvature is negative. It is not shown in Fig. 1.

Finally, Fig. 1 shows planar reaction pathways. These reaction paths are one-dimensional curves with $C_s$ symmetry connecting the reactants with the products along points of lowest energy through intermediate saddle points and local and global minima, i.e. minimum energy paths (MEPs). Two pathways connecting transition states and minima exist. The first pathway passes through the global minimum with its $D_{2h}$ symmetry and crosses over the tight TS2 transition state at $hc\times -1468\,\hbox {cm}^{-1}$ to reach the local minimum at $hc\times -2736\,\hbox {cm}^{-1}$ and the product state. The TS2 saddle point can also direct the first pathway back to the global minimum and reactant state. The second pathway has a flatter energy landscape associated with the looser saddle points labelled $D_{2h}$TS and TS1 as well as the two equivalent local minima at $hc\times -1840\,\hbox {cm}^{-1}$. Both pathways encounter our two roaming transition states.

We end this subsection by noting that when not only the positions of the atoms lie in a single plane but also their momenta are directed in this plane then there exist no forces from the interatomic potentials that can break $C_s$ symmetry. Similarly, if the positions and momenta of the four atoms lie on a single line, then they will always remain or move along this line.

### Collision time of the intermediate complex

In this subsection we determine the mean duration or mean collision time of the intermediate four-atom $\hbox {Na}_2\hbox {Rb}_2$ complex. We do so by computing an ensemble of classical trajectories of two colliding $^{23}\hbox {Na}^{87}$Rb molecules using the Mercury/Venus96 code^44,61^. For these classical simulations we use an analytical ground-state PES based on the dimer-in-molecule (DIM) approach described in Methods. Essentially, this four-atom potential is constructed from the spectroscopically-accurate singlet $\hbox {X}^1\Sigma ^+$ and triplet $\hbox {a}^3\Sigma ^+$ pair-wise potentials for each of the dimers in the complex such that the total electron spin of the four doublet alkali-metal atoms is zero. Coupled-cluster calculations, described in the previous subsection, are too computationally demanding within the Mercury/Venus96 code. The DIM potential can be quickly computed for any geometry of the four atoms, while still giving a reasonable description of the four-body potential.

We set the total energy in the classical simulations equal to that of two $^{23}\hbox {Na}^{87}$Rb molecules, each in their $v=0,J=0$ ro-vibrational state plus a fixed relative kinetic energy of $k\times 1$ mK, where *k* is the Boltzmann constant. We use micro-canonical sampling for the initial relative orientation of the molecules and initial relative momenta within each molecule given the constraints from the total energy and zero point energies of the $^{23}\hbox {Na}^{87}$Rb dimers. We mainly present data for simulations with an impact parameter equal to zero. At cold collision energies collisional lifetimes (and other “global” properties) are nearly independent of impact parameter up to impact parameters about five times larger than the equilibrium separation of ground state $^{23}\hbox {Na}^{87}$Rb. We postpone further discussion of the role of the impact parameter until the end of the last subsection of Results. Finally, the calculations are started at a molecule-molecule separation of $R_s=57 a_0$, where the potential energy between the molecules is approximately $hc\times -1\,\hbox {cm}^{-1}$. The fixed propagation time step is 0.1 fs. Our calculations show that the total energy is conserved to ${hc}{\times}0.1\, {cm}^{-1}$ on the order of the rotational constant of ${hc} {\times} 0.07 \hbox {cm}^{-1}$. This change in total energy is sufficiently small for the purposes of finding collision times and excitation rates.

**Figure 2:**
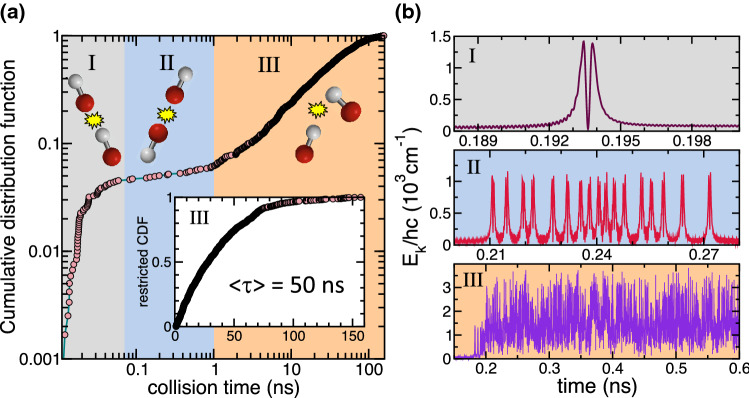
(**a**) Cumulative distribution function of the duration or collision time $\tau$ of the collisional complex in cold $^{23}\hbox {Na}^{87}$Rb+$^{23}\hbox {Na}^{87}$Rb collisions determined from classical trajectory simulations starting with zero impact parameter. For each of the approximately 1200 trajectories the initial state is sampled assuming that the molecules are in the $v=0,J=0$ ro-vibrational ground state. Three classes of trajectories are observed. They are indicated with colored bands and the roman numerals I, II, or III. The inset shows the restricted cumulative distribution function for trajectories versus $\tau$ in class III. (**b**) Total kinetic energies $E_{\mathrm{k}}$ as functions of time since the start of the simulation for one trajectory taken from each of the three classes. Figures were prepared using Microsoft Power Point for Mac version 16.47 and Grace-5.1.22 software.

For the purpose of defining the duration or lifetime of a collision, a collision starts when for the first time the total kinetic energy is larger than two times the initial total kinetic energy. It ends when for the last time the total kinetic energy is less than two times this initial total kinetic energy. The time difference is the lifetime of a trajectory $\tau$. The numerical simulations halt when the separation between products reaches $R_f = R_s-0.5a_0$ (The choice of halting separation slightly less than $R_s$ is an artifact of the fact that for cold collision and our choice of $R_s$ products can not separate to infinity. We have verified that the conclusions in this section do not change when we use initial relative kinetic energies larger than $hc\times 1\,\hbox {cm}^{-1}$.) We then analyze the geometry of the four-atom system. Mainly, the products are simply those of the initial state or those where the two sodium atoms (or the two rubidium atoms) have interchanged. In rare cases the classical simulations lead to the homonuclear $\hbox {Na}_2$+$\hbox {Rb}_2$ products. The endothermicity of the system, shown in Fig. 1, is smaller than the zero point energy of both NaRb+NaRb and $\hbox {Na}_2$+$\hbox {Rb}_2$. Thus with micro-canonical sampling the product $\hbox {Na}_2$+$\hbox {Rb}_2$ can sometimes appear. In any quantum mechanical simulation this product cannot form. They do not significantly affect our value for the collision lifetime.

Figure 2(a) shows the cumulative distribution function (CDF) of lifetimes $\tau$ as determined from approximately 1200 zero-impact-parameter trajectories on a double logarithmic scale. The cumulative distribution function at argument $\tau$ is the probability that the collision time is less than or equal to $\tau$. We observe that there exist three distinct classes of trajectories. Class I includes trajectories with $\tau <0.07$ ns. This group corresponds to approximately 4% of the trajectories. Class II includes the trajectories with $\tau$ between 0.07 ns and 1 ns and has fewer members. Class III consists of long-lived trajectories with $\tau$ larger than 1 ns with a majority of cases around 50 ns.

The inset of Fig. 2(a) depicts the cumulative distribution function for paths with $\tau > 1$ ns. This restricted CDF is well represented by the function $1-\exp (-\tau /\langle \tau \rangle )$ corresponding to an exponential or Poissonian probability function of lifetimes. In fact, the mean lifetime $\langle \tau \rangle$ is 50 ns from a least-squares fit.

Figure 2(b) shows examples of the total kinetic energy as functions of time for one trajectory out of each of the three groups. The behavior of the kinetic energy for short-lived, class-I trajectories is intriguing. An in-depth investigation showed that for most of these trajectories the two NaRb molecules approach each other in near collinear orientation and collide *head-to-tail*, as sketched in Fig. 2(a), and their motion is quasi-one-dimensional with small excursions along perpendicular directions weakly breaking collinear symmetry. Moreover, the atoms come close only once, corresponding to the near zero kinetic energy around time 0.1936 ns. The existence of these trajectories follows from the observation that when the positions and velocities of the atoms are in a single common direction the forces that would move the atoms out of alignment are zero. The relatively-large 4% probability of such collisions is due to the small positive curvature of the potentials along all transverse directions. The smaller this curvature or frequency, the larger the acceptance angle for quasi-1D collisions. The rare class-II trajectories are initially head-to-head collisions with larger rotational energy leading to their multiple re-collisions with various orientations.

Finally, in long-lived, class III collisions the atoms undergo chaotic motion with all orientations, geometries, and velocities appearing at random times. Short intervals of regular motion, however, do appear once in a while and will be discussed in the next section.

### Roaming dynamics and conical intersections

Analysis of the long-lived, class III trajectories discussed in the previous section has led us to the observation of unconventional *roaming* pathways, previously observed in unimolecular and atom-molecule reactions with light atoms^48,50,62^. A main characteristic of roaming pathways is that they go through near-threshold roaming transition states (r-TS). They exhibit bond stretching with large amplitude relative motion of molecules.

**Figure 3:**
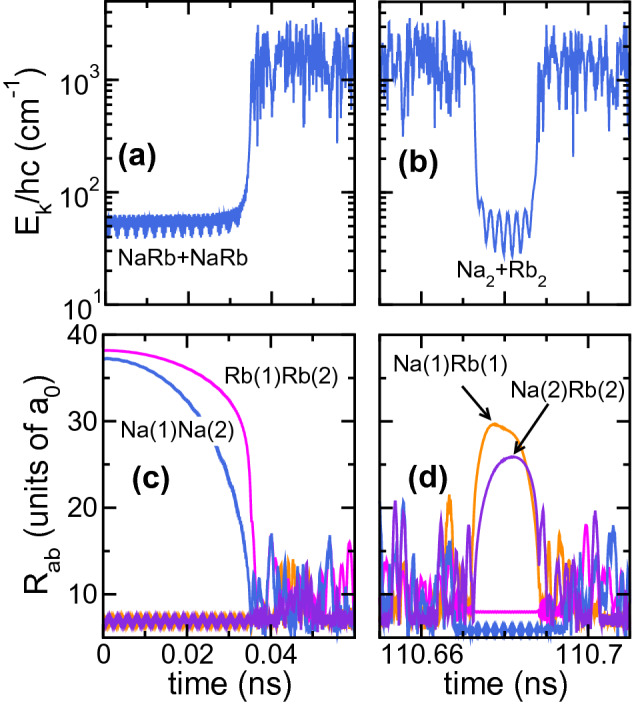
Total kinetic energy, $E_k$, and interatomic separations, $R_{ab}$, as functions of time for a zero-impact-parameter trajectory with a lifetime of 200 ns. Panel (**a**) shows $E_k$ for the first 0.06 ns of the association of two NaRb into a tetramer complex. Panel (**b**) shows a roaming event that occurs 110.66 ns into the collision. Panels (**c**) and (**d**) show the relevant pair separations between the Na and Rb atoms as functions of time for the time intervals in panels (**a**) and (**b**), respectively. Only pair separations with the largest values have been labeled. The line colors in the two panels have the same meaning. Here, roaming leads to the temporary formation of homonuclear $\hbox {Na}_2$ and $\hbox {Rb}_2$. Figures were prepared using Grace-5.1.22 software.

We observe multiple appearances of roaming pathways in the complex-forming $\hbox {Na}_2\hbox {Rb}_2$ trajectories. Motion in the complex is once in a while interrupted by dimer molecules moving away from the complex with large amplitude angular motion. This lasts for a few tens of ps after which they return to again form a four-atom intermediate. This process continues until products or reactants have enough kinetic energy to fly away from each other. An example of such event is shown in Fig. 3. Panels (a) and (b) of this figure show the total kinetic energy, $E_k$, of a long-lived trajectory with lifetime $\tau =200$ ns as functions of time. Panel (a) shows a time slice just at the beginning of the association of two NaRb into a tetramer. Panel (b) displays a roaming event in the trajectory. Panels (c) and (d) show the relevant pair separations. The bond between Na and Rb atoms is temporarily weakened, their separations are stretched out to $\approx 30 a_0$, while at the same time $\hbox {Na}_2$ and $\hbox {Rb}_2$ molecules are formed near the roaming-TS on the right-hand side of Fig. 1. These homonuclear dimers slowly roam about each other in the van der Waals region of the potential. After about seven vibrational periods of the homonuclear dimers the roaming molecules return back to reform the tetramer. In other trajectories with long lifetimes, we have also observed roaming to NaRb+NaRb over similarly brief time periods corresponding to the roaming-TS on the left-hand side of Fig. 1.

Previous studies of roaming with hydrogen-containing molecules^63,64^ emphasized the role that seams of conical intersections (CIs) between two PESs^45^ play. Here, CIs are the geometries where the potential energies of molecular states with the same properties under symmetry operations are equal. The existence of CIs in alkali-metal tetramers was previously predicted by Refs.^30,65^. For a better understanding of roaming in $\hbox {Na}_2\hbox {Rb}_2$, we studied the potentials of the singlet ground state and the first singlet excited state within the dimer-in-atom model (See Methods for the calculation of the excited-state potential.) The dimer-in-atom model puts all CIs at $D_{2h}$ geometries.

**Figure 4:**
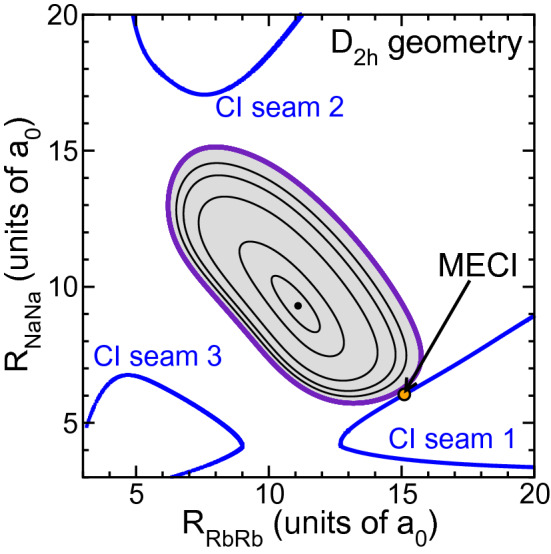
Contour plot of seams of conical intersections (thick blue curves) and potential energies (black curves) of $\hbox {Na}_2\hbox {Rb}_2$ in planar $D_{ 2h}$ symmetry as functions of separations $R_{\mathrm{NaNa}}$ and $R_{\mathrm{RbRb}}$. The orange marker indicates the minimum-energy conical intersection (MECI) on seam 1. The grey area indicates $D_{2h}$ geometries that are classically accessible for a cold NaRb+NaRb collision. Figure was prepared using Grace-5.1.22 software.

Figure 4 shows that there exist three continuous CI seams in $D_{2h}$ geometries, uniquely described by the homonuclear pair separations $R_{\mathrm{NaNa}}$ and $R_{\mathrm{RbRb}}$. Two of these seams occur when either the $R_{\mathrm{NaNa}}$ or $R_{\mathrm{RbRb}}$ is significantly larger than their dimer ground-state equilibrium separation. The CI geometry where the potential energy is lowest on any of the three seams is also shown in the figure. This minimum-energy conical intersection (MECI) is about $hc\times 500\,\hbox {cm}^{-1}$ higher in energy than our entrance-channel energy and, thus, does not overlap the classically allowed region for the quasi-classical trajectories. We must conclude that roaming does not require the presence of CIs. The complex landscape of the six-dimensional potential energy surface with its chaotic motion is sufficient for the appearance of roaming.

### Light-induced transition to excited states

In this subsection, we describe the time-dependent quasiclassical dressed-state scattering of two cold $^{23}\hbox {Na}^{87}$Rb molecules in the presence of trapping laser light with a wavelength of 1064 nm. We also estimate the probability that the laser excites the four-atom complex during the 50 ns mean collisional lifetime found in the previous subsections.

**Figure 5:**
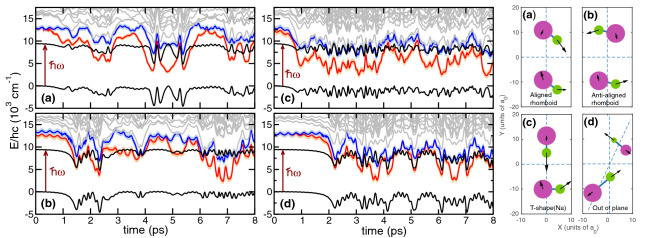
*On-the-fly* dressed-state model of optical excitations of two classically-colliding cold ground-state $^{23}\hbox {Na}^{87}$Rb molecules in the presence of an infra-red trapping laser with a wavelength of 1064 nm. Panels (a)-(d) show singlet ground- (solid black curve) and excited-states (solid red, blue, and grey curves) potential energies as functions of time during the first few ps of trajectories with zero impact parameter, but different initial relative orientations and velocities of the cold molecules. These initial orientations and velocities are shown in the four small panels on the right with corresponding panel labels. Large magenta and smaller green filled circles give the positions of Rb and Na atoms, respectively, while arrows of different lengths specify their velocities. The second higher-in-energy black curves in the panels on the left correspond to the ground-state potential plus the energy of a photon from the 1064 nm laser. Excitations occur when this dressed ground-state potential crosses that of excited states. The error bands on the potentials of the first (orange curve) and second (blue curve) excited states indicate the difference in energy splittings between excited states and the ground state as found by TD-DFT and EOM-CCSD calculations. Figures were prepared using Grace-5.1.22 software.

For these goals we do not only need the electronic ground state but also excited states that have significant electric-dipole coupling with the ground state, i.e. those that are optically-active and can be resonantly excited. The dimer-in-molecule model cannot be used to determine all relevant optically-active excited-state potentials and electric dipole moments. Instead, we use density functional theory (DFT) to obtain the *on-the-fly* ground-state potential, as the four atoms classically evolve under the forces due to this potential, and time-dependent density functional theory (TD-DFT) to compute splittings between ground- and excited-state potentials as well as transition dipole moments. A description of these electronic-structure calculations is given in Methods.

Figure 5 shows ground- and spin-singlet excited-state potential energies as functions of time up to 8 ps for four of our trajectories as well as their initial state sampled from the $v = 0, J = 0\,\hbox {X}^1\Sigma ^+$ state of NaRb. Their initial relative kinetic energy is 1 mK at an initial dimer separation of $38a_0$. The trajectories are calculated using a variable time step size no larger than 4 fs. For the trajectories the collision did not finish before we stopped the simulations. In the figure we also show the energy of the dressed ground state corresponding to the ground-state potential plus the energy of a 1064 nm photon. The four-atom complex can be optically-excited when the potential energy of an excited state equals that of the dressed ground state. In fact, for the 1064 nm laser the potential of only the two energetically lowest excited states “cross” that of the dressed ground state. The figure shows multiple crossings within our ps time windows.

The computation of *on-the-fly* DFT ground and excited potentials is numerically demanding and we had to decrease the initial dimer separation from that used in the previous sections and limit the time evolution to about 10 ps. Error bands around excited-state potential energies in Fig. 5 are our estimates of the standard-deviation uncertainty of splittings between potentials based on additional EOM-CCSD calculations for the three energetically lowest potential surfaces.

The crossings shown in Fig. 5 immediately suggest a means to determine the probability of non-adiabatic transitions between the potentials. The physics reduces to a time-dependent two-state system, considered by Landau^56^ and Zener^57^, with diabatic energies that vary linearly in time and a time-independent coupling due to the molecule-laser interaction. In the spatial domain this corresponds to a transition from one potential to another that is “vertical”, i.e. where the atomic positions and velocities are unchanged in the transition. Landau-Zener theory then states that the transition probability of excitation from the ground state at the $i^{\mathrm{th}}$ crossing, occurring at time $t_i$ and $i=1,2,3,\cdots$, is

1 $$\begin{aligned} p_{i} = \exp \left( -2\pi \frac{\Omega (t_i)^2}{\hbar |\alpha (t_i)|}\right) \,, \end{aligned}$$

where energy

2 $$\begin{aligned} \Omega (t_i) = d(t_i) \sqrt{\frac{2I}{\epsilon _0 c}} \end{aligned}$$

is the coupling matrix element that depends on transition electric dipole moment $d(t_i)$ to the relevant excited state and laser intensity *I*. Here, $\epsilon _0$ is the vacuum permittivity. The rate of change of the energies at time $t_i$ is $\alpha (t_i)=v_i \beta _i$, where $v_i$ is the velocity along the classical path $\mathbf{R}_{\mathrm{cl}}(t)$ of the atomic coordinates at $t=t_i$ and

3 $$\begin{aligned} \beta _i = \mathbf{n}_{\mathrm{cl}}\cdot \nabla \left( V_{\text {gr}}(\mathbf{R}_{\mathrm{cl}}) - V_{\text {exc}}(\mathbf{R}_{\mathrm{cl}})\right) \end{aligned}$$

is the spatial derivative of the potential energy difference between the ground- and excited-state potential, $V_{\text {gr}}(\mathbf{R})$ and $V_{\text {exc}}(\mathbf{R})$, respectively, along the classical path at $t=t_i$. Here, direction $\mathbf{n}_{\mathrm{cl}}=\mathbf{R}_{\mathrm{cl}}/|\mathbf{R}_{\mathrm{cl}}|$.

Once excited the atoms accelerate and move according to the forces generated by the electronically excited state potential. The excited state can decay back to the ground state by spontaneous emission or undergo other Landau-Zener transitions, either back to the ground state or up to doubly-excited states whenever dressed-state potentials are resonant. In either case the system’s molecular total energy has changed sufficiently from that of the initial collision state so that the molecules are no longer trapped by the laser and lost.

For our system typical values of $|\beta (t_i)|$ are $\sim hc\times 10^{-2}\ \mathrm{cm}^{-1}/a_0$, while those of $|d(t_i)|$ range from $10^{-3}\,ea_0$ to $1\,ea_0$, where *e* is the elementary charge. Moreover, for a laser wavelength of 1064 nm the mean time interval between crossings $\delta t=\langle t_{i+1}-t_i\rangle$ is 0.23 ps based on averaging within a trajectory and our sample of trajectories. In fact, we find that the intervals $t_{i+1}-t_i$ have a Poisson distribution. For a laser at this wavelength and an intensity of 10 kW/$\hbox {cm}^2$, the mean excitation probability for a crossing ${\bar{p}}=\langle p_i\rangle$ is $2.1\times 10^{-5}$, orders of magnitude smaller than one. From Eq. (1) it follows that ${\bar{p}}$ is proportional to laser intensity. The probability distributions for $t_{i+1}-t_i$ and $p_i$, which we find to be uncorrelated, are not expected to change for propagation times significantly larger than 10 ps.

As we are unable to run the *on-the-fly* calculations out to collision times of order of tens of nanoseconds, we must derive a coarse grained model for the excitation process based on the inequality $\delta t \ll \tau$. First, we realize that after *N* crossings for each trajectory the total or cumulative likelihood to be excited, and thus lost, is

4 $$\begin{aligned} \sum _{i=1}^N \left[ \prod _{j=1}^{i-1}(1-p_j)\right] p_i \,. \end{aligned}$$

This corresponds to time $t=t_N-t_1=\sum _{i=1}^{N-1}(t_{i+1}-t_i)$ after the first excitation. Next, we assume that $t\gg \delta t$ (and thus $N\gg 1$) and sample $p_i$ and $t_{i+1}-t_i$ from their respective probability distributions. The averaged cumulative excitation probability at time *t* is then well approximated by

5 $$\begin{aligned} P(t)\approx & {} 1 - e^{-{\bar{p}} t/\delta t} \equiv 1 - e^{- \kappa t} \end{aligned}$$

by replacing $p_i$ and $t_{i+1}-t_i$ by their mean values, using the geometric series to evaluate the finite sum, and using that ${\bar{p}}\ll 1$. On the right hand side of the equation, we defined the physically relevant laser-induced survival rate $\kappa ={\bar{p}}/\delta t$ of the collisional complex, which for typical laser intensities is proportional to the laser intensity.

For the laser with a wavelength of 1064 nm and an intensity of 10 kW/$\hbox {cm}^2$ the decay time $1/\kappa = 12$ ns, much larger than $\delta t$, justifying our derivation. On the other hand the 12-ns decay time is on the order of the mean lifetime $\langle \tau \rangle$ of the collisional complex. These two characteristic times of the collisional complex can be combined to give the likelihood that a NaRb molecule does not survive the collision. This likelihood is $P(\langle \tau \rangle )$, the averaged cumulative excitation probability at time $\langle \tau \rangle$. For a 1064 nm laser at ${I=10}$ kW/$\hbox {cm}^2$ its value is ${1-0.016}$, close to one. A NaRb molecule is not likely to survive a collision in the presence of 1064 nm light.

Finally, we make some observations regarding the relevance of the impact parameter on our conclusions so far. All reported data have been for zero impact parameter. From additional trajectory simulations (not shown) we found that for impact parameters as large as $40a_0$, approximately five times the di-atomic X-state equilibrium separations, the duration of the collision and mean excitation probabilities fluctuate by no more than 20%. (Impact parameters larger than $40a_0$ are inconsistent with our initial molecular separation and have not been considered.) The independence of the relevant quantities with respect to impact parameter is consistent with the theory behind spiraling collisions, Langevin or orbiting cross sections, as well as the chaotic motion in the six-dimensional ground-state potential. Firstly, because for low collision energies and the attractive long-range van-der-Waals interaction between two NaRb molecules, the maximum expected contributing impact parameter^66^ is several times larger than $40a_0$. Moreover, chaotic motion with its inherent sampling of all allowed phase space implies that once the short-ranged four-body complex is formed at the beginning of the collision the typical or mean collision time is independent of impact parameter. Thus, we can conclude that the light-induced inelastic cross section is approximately $P(\langle \tau \rangle )$ times the Langevin cross section.

## Discussion

We have performed a detailed examination of classical collision trajectories in $^{23}\hbox {Na}^{87}$Rb+$^{23}\hbox {Na}^{87}$Rb interactions both with and without the presence of laser light trapping the cold molecules. We computed the mean collision duration of the four-body complex without laser excitations and showed evidence of a long-lived “collision complex” that includes roaming motion to briefly form homonuclear $^{23}\hbox {Na}_2$+$^{87}\hbox {Rb}_2$. We also determined the survival or collision time of the four-body complex in the presence of 1064-nm laser light, which can induce transitions to two electronically excited states, and found that it is of the same order of magnitude as the duration of the four-body complex for a typical experimental laser intensity of 10 kW/$\hbox {cm}^2$. Under these circumstances $^{23}\hbox {Na}^{87}$Rb molecules will be rapidly removed from the optical trap.

Our investigation, which includes roaming pathways within the collisional complex, leads to different, much-shorter lifetimes than those from the RRKM theory, which does not have a concept of roaming pathways. We believe that the existence of roaming events during collisions can significantly modify the collisional lifetime and change the reaction outcome. One example of such effect is given in Ref.^67^, which reported the several orders of magnitude decrease of the rate constant for H-atom formation in photodissociation of $\hbox {C}_2\hbox {H}_5$ molecule due to the roaming dissociation channel.

Recent experiments^37,38^, using chopped ODTs with a time duration of laser pulses of order of 100 µs and/or box-shaped potentials, attempted to decrease the molecular loss rate. No change was observed. Our calculations giving short collision times of 50 ns would predict that colliding molecules are excited by the trapping light and mostly lost from the trap. Other loss mechanisms must be present when the laser light is not present.

Several open questions remain regarding the validity of the approximations used in the paper and must be answered with future investigations. These concerns include the need for full quantum simulations of the collision on the ground-state potential, treatment of the CIs, and more sophisticated models for laser excitations. We also envision a study of a wavelength dependence of the optical excitation. Longer wavelengths should lead to fewer excitations as, for example, excitations to the second excited electronic state become energetically forbidden. Those to the first excited state with its CIs with the ground state potential are always possible. Nevertheless, we feel that the general conclusions will not be invalidated.

## Methods

### Dimer-in-molecule potentials

For the determination of the mean duration of $^{23}\hbox {Na}^{87}$Rb+$^{23}\hbox {Na}^{87}$Rb collisions and the tetramer conical intersections we require an easy to compute but at the same time reasonably accurate electron-spin-singlet ground-state PES. We use the six-dimensional non-relativistic potential based on the dimer-in-molecule theory of Ref.^43^. In this theory the electron wavefunctions of the tetramer are approximated by superpositions of products of four spin-1/2 ground-state alkali-metal-atom electron wavefunctions, such that the total electron spin is zero. Two (geometry- or position-independent) singlet electron wavefunctions can be constructed with straight-forward application of angular momentum algebra^68^. The corresponding $2\times 2$ tetramer potential matrix is determined from matrix elements for the sum of six pairwise potential operators. Each pairwise potential operator is given by $\sum _{S_{ij}=0}^1 P_{S_{ij}} V_{S_{ij}}(R_{ij})$, where $S_{ij}$ is the electron spin of atom pair (*i*, *j*), operator $P_{S_{ij}}$ is the projection operator on states with pair spin $S_{ij}$ with $P_0+P_1$ equal to the identity operator, and $V_{S_{ij}}(R_{ij})$ is the isotropic pair-wise $\hbox {X}^1\Sigma ^+$ (or $\hbox {X}^1\Sigma ^+_g$) Born-Oppenheimer potential at pair separation $R_{ij}$ for $S_{ij}=0$ and the triplet $\hbox {a}^3\Sigma ^+$ (or $\hbox {a}^3\Sigma ^+_u$) potential for $S_{ij}=1$. The three matrix elements of the $2\times 2$ matrix are sums of $V_{S_{ij}}(R_{ij})$ with weights given by matrix elements of a unitary transformation reflecting the different ways to couple four spin-1/2 atoms into pairs. These weights are proportional to a nine-*j* symbol and phase factors^68^.

We use the spectroscopically-accurate dimer potentials from Refs.^69–71^ with adjustments to their repulsive walls at small pair separations, where the potential energy is larger than that at the dissociation limit. These adjustments removed unphysical trends going from the light $\hbox {Na}_2$, to NaRb, to the heavier $\hbox {Rb}_2$ dimers. The extrapolation of spectroscopic data to small separations can not reliably specify the shape of the inner walls of potentials.

The energetically-lowest eigenvalue of the $2\times 2$ potential matrix corresponds to the ground-state tetramer potential and is used in classical trajectory calculations. This PES is not separable but, nevertheless, does not include so-called non-additive contributions. We assume that the effects of such contributions on the mean lifetime are small. Here, we rely on the results of time-independent quantum-mechanical studies of reactive atom-dimer collisions with alkali-metal atoms and molecules^72,73^. They found that total reaction rate coefficients are not affected when the non-additive part of the potential is or is not included. Similarly, we assume that the lifetimes are not affected by the precise nature of the adjustments to the inner walls of the pair potentials. In addition, long-range four-body electric dipole-dipole and quadrupole-quadrupole interactions between the polar NaRb molecules are absent in our PES. We do note that our cold entrance channel NaRb+NaRb conditions only include the non-rotating J=0 ground state only. Then to first order these four-body interactions are averaged out and should not affect our estimated value of the collision time.

Conical intersections in $2\times 2$ dimer-in-molecule model occur when the two eigenvalues of the matrix are equal. A necessary condition for such occurrence is that the off-diagonal matrix element is zero. Inspection of the corresponding weighted sum of $V_{S_{ij}}(R_{ij})$ for $\hbox {Na}_2\hbox {Rb}_2$ shows that this matrix element is zero for all geometries with $D_{2h}$ symmetry. Thus, for such geometries conical intersections occur when the diagonal elements of the $2\times 2$ matrix are equal. Figure 4 shows the locations of the conical intersections in the dimer-in-molecule model.

### The *on-the-fly* optical excitation model

The *on-the-fly* optical excitation model for $^{23}\hbox {Na}^{87}$Rb+$^{23}\hbox {Na}^{87}$Rb collisions in the presence of a laser field has two steps. First, we performed classical trajectory calculations using the *on-the-fly* ground-state electronic potential energies and gradients from a density functional theory (DFT) method. Second, at each of the geometries along a trajectory we applied time-dependent density functional theory (TD-DFT) calculations to obtain energy splittings between ground and electronically excited states as well as the corresponding electric dipole moment. The molecular dynamics equations have only been propagated to about 10 ps.

The DFT calculations have been performed for total electron spin ${S=0}$ using the hybrid functional wb97xd^74^. This functional is a long-range corrected functional that allows for a good description of the long-range van-der-Waals interaction between atoms and molecules. We use a correlation-consistent triple zeta basis (cc-pvtz) for the Na atom and the Stuttgart/Cologne-group effective-core potential ECP28MDF^75^ for the Rb atom with Hill and Peterson’s augmented correlation-consistent quadruple-zeta polarization potential basis (aug-cc-pvqz-pp)^76^. This choice of basis allows for reasonable computational times of the *on-the-fly* dynamics.

The TD-DFT calculations, which found about ten excited states, are based on the spin-unrestricted Coulomb-attenuated B3LYP functional (uCAM-B3LYP). We compare the lowest two TD-DFT excitation energies with those from coupled-cluster equation-of-motion with singles and doubles (EOM-CCSD) calculations using the same basis set and find a nearly 5% difference as shown in Fig. 5. This small difference is sufficient for our purposes.

## References

[1] Ni K-K (2008). A high phase-space-density gas of polar molecules. Science.

[2] Liu Y (2020). Photo-excitation of long-lived transient intermediates in ultracold reactions. Nat. Phys..

[3] Takekoshi T (2014). Ultracold dense samples of dipolar RbCs molecules in the rovibrational and hyperfine ground state. Phys. Rev. Lett..

[4] Molony PK (2014). Creation of ultracold $$^{87}{\rm Rb}^{133}{\rm Cs}$$ molecules in the rovibrational ground state. Phys. Rev. Lett..

[5] Park JW, Will SA, Zwierlein MW (2015). Ultracold dipolar gas of fermionic $$^{23}{\rm Na}^{40}{\rm K}$$ molecules in their absolute ground state. Phys. Rev. Lett..

[6] Guo MY (2016). Creation of an ultracold gas of ground-state dipolar $$^{23}{\rm Na}^{87}{\rm Rb}$$ molecules. Phys. Rev. Lett..

[7] Seeßelberg F (2018). Modeling the adiabatic creation of ultracold polar $$^{23}{\rm Na}^{40}{\rm K}$$ molecules. Phys. Rev. A.

[8] Voges KK (2020). Ultracold gas of bosonic $$^{23}{\rm Na}^{39}{\rm K}$$ ground-state molecules. Phys. Rev. Lett..

[9] Chen K (2011). Molecular-ion trap-depletion spectroscopy of BaCl$${}^{+}$$. Phys. Rev. A.

[10] Halvick P, Stoecklin T, Lique F, Hochlaf M (2011). Explicitly correlated treatment of the Ar-NO$$^{+}$$ cation. J. Chem. Phys..

[11] Sullivan ST, Rellergert WG, Kotochigova S, Hudson ER (2012). Role of electronic excitations in ground-state-forbidden inelastic collisions between ultracold atoms and ions. Phys. Rev. Lett..

[12] Rellergert WG (2013). Evidence for sympathetic vibrational cooling of translationally cold molecules. Nature.

[13] Härter A (2013). Population distribution of product states following three-body recombination in an ultracold atomic gas. Nat. Phys..

[14] Stoecklin T (2016). Explanation of efficient quenching of molecular ion vibrational motion by ultracold atoms. Nat. Commun..

[15] Pérez-Ríos J (2019). Vibrational quenching and reactive processes of weakly bound molecular ions colliding with atoms at cold temperatures. Phys. Rev. A.

[16] Dörfler AD (2020). Rotational-state-changing collisions between N$$_{2}^{+}$$ and Rb at low energies. Phys. Rev. A.

[17] Mohammadi A (2021). Life and death of a cold BaR$$\text{b}^{+}$$ molecule inside an ultracold cloud of Rb atoms. Phys. Rev. Res..

[18] Krems RV (2008). Cold controlled chemistry. Phys. Chem. Chem. Phys..

[19] Bell, M. T. & Softley, T. P. Ultracold molecules and ultracold chemistry. *Mol. Phys.* **107**, 99–132 (2009).

[20] Dulieu O, Krems R, Weidemüller M, Willitsch S (2011). Physics and chemistry of cold molecules. Phys. Chem. Chem. Phys..

[21] Balakrishnan N (2016). Perspective: Ultracold molecules and the dawn of cold controlled chemistry. J. Chem. Phys..

[22] Hu M-G (2019). Direct observation of bimolecular reactions of ultracold $$\rm KRb$$ molecules. Science.

[23] Hu M-G (2020). Nuclear spin conservation enables state-to-state control of ultracold molecular reactions. Nat. Chem..

[24] Micheli A, Brennen GK, Zoller P (2006). A toolbox for lattice-spin models with polar molecules. Nat. Phys..

[25] Yan B (2013). Observation of dipolar spin-exchange interactions with lattice-confined polar molecules. Nature.

[26] DeMille D (2002). Quantum computation with trapped polar molecules. Phys. Rev. Lett..

[27] Ni K-K, Rosenband T, Grimes DD (2018). Dipolar exchange quantum logic gate with polar molecules. Chem. Sci..

[28] Sawant R (2020). Ultracold polar molecules as qudits. New J. Phys..

[29] Hughes M (2020). Robust entangling gate for polar molecules using magnetic and microwave fields. Phys. Rev. A.

[30] Żuchowski PS, Hutson JM (2010). Reactions of ultracold alkali-metal dimers. Phys. Rev. A.

[31] Ospelkaus S (2010). Quantum-state controlled chemical reactions of ultracold potassium-rubidium molecules. Science.

[32] Idziaszek Z, Julienne PS (2010). Universal rate constants for reactive collisions of ultracold molecules. Phys. Rev. Lett..

[33] Kotochigova S (2010). Dispersion interactions and reactive collisions of ultracold polar molecules. New J. Phys..

[34] De Marco L (2019). A degenerate Fermi gas of polar molecules. Science.

[35] Ye X, Guo M, González-Martínez ML, Quéméner G, Wang D (2018). Collisions of ultracold $$^{23}{\rm Na}^{87}{\rm Rb}$$ molecules with controlled chemical reactivities. Sci. Adv..

[36] Gregory PD (2019). Sticky collisions of ultracold $${\rm RbCs}$$ molecules. Nat. Commun..

[37] Bause, R. *et al.* Collisions of ultracold molecules in bright and dark optical dipole traps. *arXiv preprint* arXiv:2103.00889v2 (2021).

[38] Gersema, P. *et al.* Probing photoinduced two-body loss of ultracold non-reactive bosonic $$^{23}$$N$$\text{ a}^{87}$$Rb and $$^{23}$$N$$\text{ a}^{39}$$K molecules. *arXiv preprint* arXiv:2103.00510v2 (2021).

[39] Christianen A, Zwierlein MW, Groenenboom GC, Karman T (2019). Photoinduced two-body loss of ultracold molecules. Phys. Rev. Lett..

[40] Gregory PD, Blackmore JA, Bromley SL, Cornish SL (2020). Loss of ultracold $$^{87}{\rm Rb}^{133}{\rm Cs} $$ molecules via optical excitation of long-lived two-body collision complexes. Phys. Rev. Lett..

[41] Bell MT, Softley TP (2009). Ultracold molecules and ultracold chemistry. Mol. Phys..

[42] Levine RD (2005). Molecular Reaction Dynamics.

[43] Ellison, F. A method of diatomics in molecules. I. general theory and application to H$$_2$$O. *J. Am. Chem. Soc.* **85**, 3540–3544 (1962).

[44] Hase WL (1996). MERCURY: A general Monte Carlo classical trajectory computer program. QCPE Bull..

[45] Domcke, W., Yarkony, D. & Köppel, H. *Conical Intersections: Theory, Computation and Experiment*, vol. 17 of *Advanced series in physical chemistry* (World Scientific, Singapore, 2011).

[46] Bowman JM (2014). Roaming. Mol. Phys..

[47] Townsend D (2004). The roaming atom: Straying from the reaction path in formaldehyde decomposition. Science.

[48] Christoffel K, Bowman J (2009). Three reaction pathways in the H + HCO $$\rightarrow $$ H$$_2$$ + CO reaction. J. Phys. Chem. A.

[49] Takayanagi T, Tanaka T (2011). Roaming dynamics in the MgH + H $$\rightarrow $$ Mg + H$$_2$$ reaction: Quantum dynamics calculations. Chem. Phys. Lett..

[50] Li A, Li J, Guo H (2013). Quantum manifestation of roaming in H+ MgH $$\rightarrow $$ Mg + H$$_2$$: The birth of roaming resonances. J. Phys. Chem. A.

[51] Quinn MS (2020). Rotating resonances in the H$$_2$$CO roaming reaction are revealed by detailed correlations. Science.

[52] Suits AG (2008). Roaming atoms and radicals: A new mechanism in molecular dissociation. Acc. Chem. Res..

[53] Herath N, Suits AG (2011). Roaming radical reactions. J. Phys. Chem. Lett..

[54] Bowman JM, Shepler BC (2011). Roaming radicals. Annu. Rev. Phys. Chem..

[55] Pratihar S, Ma X, Homayoon Z, Barnes GL, Hase WL (2017). Direct chemical dynamics simulations. J. Am. Chem. Soc..

[56] Landau LD (1932). Zur theorie der Energieübertragung II. Z. Sowjetunion.

[57] Zener C (1932). Non-adiabatic crossing of energy levels. R. Soc. Lond. A.

[58] Weigend F, Ahlrichs R (2005). Balanced basis sets of split valence, triple zeta valence and quadruple zeta valence quality for H to Rn: Design and assessment of accuracy. Phys. Chem. Chem. Phys..

[59] Byrd JN, Harvey Michels H, Montgomery JA, Cote R, Stwalley WC (2012). Structure, energetics, and reactions of alkali tetramers. J. Chem. Phys..

[60] Harding LB, Klippenstein SJ, Jasper AW (2012). Separability of tight and roaming pathways to molecular decomposition. J. Phys. Chem. A.

[61] Garrido JD, Ellakkis S, Ballester MY (2019). Relaxation of vibrationally excited OH radical by SO. J. Phys. Chem. A.

[62] Bowman JM, Suits AG (2011). Roaming reactions: The third way. Phys. Today.

[63] Nakamura M (2014). Dynamical, spectroscopic and computational imaging of bond breaking in photodissociation: Roaming and role of conical intersections. Farad. Discus..

[64] Maeda S, Taketsugu T, Ohno K, Morokuma K (2015). From roaming atoms to hopping surfaces: Mapping out global reaction routes in photochemistry. J. Am. Chem. Soc..

[65] Wallis AOG, Gardiner SA, Hutson JM (2009). Conical intersections in laboratory coordinates with ultracold molecules. Phys. Rev. Lett..

[66] Child MS (1974). Molecular collision theory.

[67] Matsugi A (2013). Roaming dissociation of ethyl radicals. J. Phys. Chem. Lett..

[68] Brink DM, Satchler GR (1993). Angular Momentum.

[69] Knoop S (2011). Feshbach spectroscopy and analysis of the interaction potentials of ultracold sodium. Phys. Rev. A.

[70] Strauss C (2010). Hyperfine, rotational, and vibrational structure of the $${a}^{3}{\Sigma }_{u}^{+}$$ state of $$^{87}{\rm Rb}_{2}$$. Phys. Rev. A.

[71] Wang F, Xiong D, Li X, Wang D, Tiemann E (2013). Observation of feshbach resonances between ultracold Na and Rb atoms. Phys. Rev. A.

[72] Croft JFE (2017). Universality and chaoticity in ultracold K+KRb chemical reactions. Nat. Commun..

[73] Kendrick BK (2021). Non-adiabatic quantum interference in the ultracold Li + LiNa $$\rightarrow $$ L$$\text{ i}_2$$ + Na reaction. Phys. Chem. Chem. Phys..

[74] Chai J-D, Head-Gordon M (2008). Long-range corrected hybrid density functionals with damped atom-atom dispersion corrections. Phys. Chem. Chem. Phys..

[75] Lim IS, Schwerdtfeger P, Metz B, Stoll H (2005). All-electron and relativistic pseudopotential studies for the group I element polarizabilities from K to element 119. J. Chem. Phys..

[76] Hill JG, Peterson KA (2017). Gaussian basis sets for use in correlated molecular calculations. XI. Pseudopotential-based and all-electron relativistic basis sets for alkali metal (K-Fr) and alkaline earth (Ca-Ra) elements. J. Chem. Phys..

